# Analysis of multiplex pathogen sentinel surveillance results of SARS-CoV-2 and influenza virus in Sichuan Province, 2024–2026

**DOI:** 10.3389/fpubh.2026.1961515

**Published:** 2026-10-20

**Authors:** Fengmiao Hu, Xingyu Zhou, Lijun Zhou, Zhirui Li, Shuang Dong, Chongkun Xiao

**Affiliations:** Sichuan Center for Disease Control and Prevention, Chengdu, Sichuan Province, China

**Keywords:** acute respiratory infection, influenza virus, respiratory coinfection, SARS-CoV-2, seasonal asynchrony, sentinel surveillance, Sichuan province

## Abstract

**Objective:**

To analyze the epidemiological characteristics and coinfection of SARS-CoV-2 and influenza virus among acute respiratory infection (ARI) cases in Sichuan Province from August 2024 to July 2026.

**Methods:**

Using 24-month surveillance data from seven national respiratory pathogen sentinel hospitals in Sichuan Province, respiratory specimens were tested for both viruses by multiplex real-time RT-PCR. Positive rates and 95% CIs were calculated by the Wilson score method; group comparisons used the chi-square test or Fisher’s exact test; independent risk factors were identified by multivariable logistic regression.

**Results:**

A total of 22,436 valid cases were included. The positive rates of SARS-CoV-2, influenza virus, and coinfection were 4.39% (95% CI: 4.13–4.67%), 9.83% (95% CI: 9.45–10.22%), and 0.05% (95% CI: 0.03–0.09%), respectively. The two viruses peaked in different seasons, with SARS-CoV-2 in summer and influenza in winter–spring. Summer was an independent risk factor for SARS-CoV-2 infection (OR = 23.88, 95% CI: 17.04–33.46); children aged 6–14 years had the highest risk of influenza (OR = 2.52, 95% CI: 2.21–2.88). The observed coinfection rate was far lower than the expectation under independent circulation of the two viruses (0.43%; O/E = 0.11, *p* < 0.001), indicating a negative (asynchronous) association rather than synergy, with marked spatiotemporal clustering.

**Conclusion:**

SARS-CoV-2 and influenza virus circulated in pronounced seasonal asynchrony in Sichuan Province, with no population-level evidence of synergy; coinfection was rare but locally clustered, supporting differentiated prevention and control strategies. Given the hospital-based sentinel design, extrapolation to the whole province should be made with caution.

## 1 Introduction

SARS-CoV-2 and influenza virus are the two most common pathogens of acute respiratory infections (ARIs). Influenza virus causes approximately 290,000–650,000 respiratory-associated deaths globally each year, and influenza-associated excess respiratory deaths in China amount to approximately 88,000 annually, representing a substantial disease burden (1, 2). Although COVID-19 has entered a phase of routine prevention and control in China since SARS-CoV-2 triggered the global pandemic in late 2019 (3), the continuous emergence of new Omicron subvariants means that the epidemic situation remains dynamic (4). Non-pharmaceutical interventions (NPIs) during the COVID-19 pandemic once reduced influenza activity to historically low levels, after which the timing and intensity of influenza epidemics were substantially altered as control measures were relaxed (5, 6), and coinfection with SARS-CoV-2 and influenza has been associated with higher risks of mechanical ventilation and death (7, 8). In this context, sustained synchronous surveillance of the two viruses is of major public health importance.

National multi-pathogen sentinel surveillance has shown marked geographical heterogeneity in the aetiological spectrum of acute respiratory infections (9); in the post-pandemic period, many parts of the country have also reported a reshaping of the circulation rhythm of SARS-CoV-2 and influenza virus compared with the pre-pandemic era (10). Sichuan Province is located in southwest China with a population of over 80 million and a humid subtropical monsoon climate; frequent population movement and labour export, together with dense primary and secondary schools and childcare facilities, create conditions for year-round transmission of respiratory infectious diseases. However, previous surveillance in the province was mostly single-pathogen or short-term special investigations, and no province-wide data have continuously covered the post-pandemic period with simultaneous dual-pathogen testing; the circulation rhythm, population and regional distribution, and coinfection characteristics of the two viruses in Sichuan remain unclear (11). Using 24 consecutive months of simultaneous SARS-CoV-2 and influenza virus surveillance data from seven national sentinel hospitals across the province (August 2024 to July 2026), this study analysed the epidemic trends, population and regional distribution, and coinfection characteristics of the two viruses, focusing on changes in their circulation rhythm in the post-pandemic period, differences in risk factors across population and seasonal dimensions, and whether coinfection entails synergistic risk and spatiotemporal clustering. Compared with previous single-year or single-city reports, continuous multi-sentinel dual-pathogen surveillance better reflects stable seasonal patterns, and the findings are more relevant to local prevention and control practice in Sichuan.

## 2 Materials and methods

### 2.1 Surveillance design

This study was a retrospective study combining descriptive and analytical components, using data from consecutively registered cases in the national respiratory pathogen sentinel surveillance network of Sichuan Province. According to the Technical Protocol for Sentinel Surveillance of Acute Respiratory Infectious Diseases (Trial) issued by the Chinese Center for Disease Control and Prevention, seven national respiratory pathogen sentinel hospitals were selected in Sichuan Province, located in Chengdu, Dazhou, Nanchong, Mianyang, Panzhihua, Zigong and Ya’an, covering the main geographical regions of the province, including the Chengdu Plain (Chengdu, Ya’an), northeastern Sichuan (Dazhou, Nanchong), northern Sichuan (Mianyang), southern Sichuan (Zigong) and the Panxi area (Panzhihua) (12).

All seven sentinel hospitals were tertiary (grade A) general hospitals located in the urban centres of their respective cities. Outpatient surveillance enrolled influenza-like illness (ILI) cases, and inpatient surveillance enrolled cases meeting the severe acute respiratory infection (SARI) case definition, representing patients attending tertiary urban medical institutions. During the surveillance period, ILI and SARI cases accounted for 66.7 and 33.3% of the total, respectively, covering both mild outpatients and severe inpatients; however, the proportion of hospitalized cases may be influenced by admission capacity and health-care-seeking habits, potentially introducing representativeness bias in case type. Owing to the sentinel network layout, the surveillance population was dominated by urban patients, with relatively insufficient coverage of rural populations and community asymptomatic cases, which may introduce health-care-seeking bias (see Limitations).

### 2.2 Study population and case definitions

ARI cases monitored by the seven sentinel hospitals from August 2024 to July 2026 were included, comprising outpatient ILI cases and inpatient SARI cases. This period followed the de-escalation of COVID-19 control measures, when population antibody levels were gradually waning after natural infection and vaccination and no large-scale interventions interfered with natural circulation, making it suitable for observing the true seasonal rhythm of the two viruses.

*ILI case definition*: outpatient or emergency visit within 3 days of illness onset, with fever (temperature ≥38 °C) accompanied by cough or sore throat.

*SARI case definition*: hospitalized patients with acute onset, a history of fever (temperature ≥38.0 °C) at onset, cough, and duration of the current respiratory infection not exceeding 10 days, identified at admission or within 48 h of admission.

### 2.3 Exclusion criteria

To ensure data quality, the following exclusion criteria were applied: (1) cases with missing SARS-CoV-2 or influenza virus nucleic acid test results; (2) cases with missing and unverifiable key demographic information (sex, age); (3) cases with obvious logical errors in time information such as collection or onset dates; (4) duplicate submissions of the same case (deduplicated by specimen number, retaining the first submission); (5) cases with abnormal test results that could not be verified or corrected against original records; and (6) cases submitted by institutions other than the seven national sentinel hospitals in this study. A total of 25,624 records were exported from the surveillance system within the window of August 2024–July 2026; after verification against the above criteria, 3,188 records were excluded and 22,436 valid cases were finally included.

### 2.4 Laboratory testing

Throat swabs, nasal swabs and other respiratory specimens were collected, stored at 2–8 °C, and transported to the network laboratory within 48 h, avoiding repeated freezing and thawing. Detection was performed using a multiplex nucleic acid detection kit for respiratory pathogens (manufacturer: CapitalBio; medical device registration certificate obtained from the National Medical Products Administration) based on a microfluidic chip multiplex real-time RT-PCR platform, with a limit of detection (LoD) meeting the requirements of industry standards for respiratory pathogen nucleic acid testing. The kit simultaneously covers 17 respiratory pathogens, including *Haemophilus influenzae*, *Streptococcus pneumoniae*, rhinovirus and respiratory syncytial virus, and can distinguish influenza A subtypes H3 and H1N1 (2009) and influenza B lineages Victoria and Yamagata; this study focused on SARS-CoV-2 and influenza virus. SARS-CoV-2 was targeted by the ORF1ab and N genes, and influenza virus by the conserved M/NP gene. Positive criteria: Ct < 37 was considered positive; Ct ≥ 40 negative; samples with 37 ≤ Ct < 40 were considered borderline and retested with the original specimen; a retest Ct ≥ 40 was considered negative, and samples remaining in the borderline range after retesting were regarded as suspicious and excluded. Positive, negative and blank controls were included in each batch for internal quality control, and the laboratory regularly participated in national and provincial external quality assessments for respiratory pathogen nucleic acid testing, all with satisfactory results.

Influenza virus nucleic acid positivity was determined by a composite rule: positivity for any of the influenza A universal, A(H3) or A(H1N1)pdm09 targets was classified as influenza A positive, and positivity for any of the influenza B universal, B/Victoria or B/Yamagata targets as influenza B positive; positivity for either influenza A or B was classified as influenza virus nucleic acid positive. A specimen in which both SARS-CoV-2 and influenza virus nucleic acids were detected was defined as a coinfection case.

### 2.5 Data management and quality control

Surveillance data were reported by each sentinel hospital through the national sentinel surveillance information system for acute respiratory infectious diseases, and a uniformly coded analytical database was established after export. Variable consistency and logic checks were performed for sex, age, region, case type, sampling date and all nucleic acid test results; abnormal records were returned to the original registration for verification and correction. Deduplication was performed using the specimen number as the unique identifier, retaining the first submission. After verification, a closure check comparing the monthly total with the overall total was performed, and analysis proceeded only after the two were confirmed consistent. All variables were coded and missing values flagged before analysis; missing data were not imputed, and complete-case analysis was performed after exclusion according to the criteria in section 2.3. Replicable scripts and intermediate results were retained throughout the analysis. The complete analysis code is provided as Supplementary material S1 and is also available from the corresponding author upon reasonable request.

### 2.6 Statistical analysis

Data processing and statistical analysis were performed using Python 3.13 (pandas, NumPy, SciPy). Count data were described by case numbers and positive rates; positive rates and their 95% CIs were calculated by the Wilson score method; odds ratios (ORs) and 95% CIs for binary variables were estimated by the Wald method from multivariable logistic regression coefficients. Comparisons of positive rates between groups used the chi-square test, with Fisher’s exact test when any expected cell count was <5. Seasons were grouped by calendar month: March to May as spring, June to August as summer, September to November as autumn, and December to February as winter. Pairwise comparisons among age groups (10 pairs) used the chi-square test with Bonferroni correction (*α′* = 0.005). To evaluate whether coinfection deviated from random independence, the observed number of coinfections was compared with the theoretical expectation under the assumption of independence of the two viruses; the expected number was calculated as *E* = N × p1 × p2, where N is the total number of monitored cases and p1 and p2 are the overall positive rates of SARS-CoV-2 and influenza virus, respectively. Under the independence assumption, the number of coinfections follows a binomial distribution with parameters (*N*, p1 × p2); because coinfection is a rare event (expected proportion ≈0.43% under independence, observed 0.05%), the exact binomial test was used instead of a normal-approximation method, which is inaccurate for rare events; the large total sample size (*N* = 22,436) ensures the binomial model is well defined, and the exact binomial test yielded the *p* value and the observed/expected ratio (O/E). A month with a SARS-CoV-2 positive rate >5% and an influenza virus positive rate >10% was defined as a co-circulation month, and the remaining months were classified as the off-peak period; coinfection rates were compared between the two periods and ORs calculated. Multivariable binary logistic regression was used to identify factors associated with SARS-CoV-2 and influenza virus infection; potential confounders (age, sex, season, case type and region) were predetermined based on the literature and a causal inference framework and were all entered into the model without stepwise selection to avoid data-driven variable selection; variance inflation factor (VIF) testing showed all VIFs <3, indicating no significant multicollinearity; reference categories were set as 0–5 years, female, winter, ILI and Nanchong, respectively; subtype proportions were calculated using influenza virus nucleic acid-positive cases as the denominator. All tests were two-sided, and *p* < 0.05 was considered statistically significant.

### 2.7 Ethics statement

This study was a retrospective data analysis based on the sentinel surveillance information system. Personal identifiers, including names, national identity card numbers, contact details and detailed addresses, were removed before data export, retaining only variables required for analysis such as age, sex, region, case type and dates; no identifiable individual information was included. The study protocol was approved by the Ethics Review Committee of Sichuan Center for Disease Control and Prevention (approval number: SCCDCIRB No. 2026-026), and informed consent was waived.

## 3 Results

### 3.1 Overall detection

A total of 25,624 records were exported from the surveillance system during August 2024–July 2026; after excluding 0 records outside the window, 3,188 records submitted by institutions other than the seven national sentinel hospitals, and 0 records with missing test results, 22,436 valid cases were included after deduplication and completeness verification, with the monthly total (22436) consistent with the overall total; among them, there were 14,970 ILI cases (66.7%) and 7,466 SARI cases (33.3%).

Among the 22,436 valid cases, 986 were positive for SARS-CoV-2, with an overall positive rate of 4.39% (95% CI: 4.13–4.67%); 2,205 were positive for influenza virus, with an overall positive rate of 9.83% (95% CI: 9.45–10.22%); 11 cases were positive for both viruses (coinfection), with a coinfection rate of 0.05% (95% CI: 0.03–0.09%).

The multiplex nucleic acid detection kit simultaneously detects multiple respiratory pathogens. Besides SARS-CoV-2 and influenza virus, other major pathogens detected during the window included *Haemophilus influenzae* (15.59%), *Streptococcus pneumoniae* (10.98%), rhinovirus (8.79%) and respiratory syncytial virus (4.15%), with *H. influenzae* and *S. pneumoniae* rates both higher than those of influenza virus (9.83%).

### 3.2 Population distribution

By sex, the SARS-CoV-2 positive rate was 3.89% (95% CI: 3.56–4.26%) among 11,788 males and 4.95% (95% CI: 4.55–5.38%) among 10,648 females; the rate was slightly higher in females, and the difference was statistically significant (*χ^2^* = 14.83, *p* < 0.001). The influenza positive rates in males and females were 9.67 and 10.00%, respectively, with no statistically significant difference (*χ^2^* = 0.69, *p* = 0.41).

Positive rates differed significantly across age groups (Table 1; Figure 1). SARS-CoV-2 was highest in the 15–34 years group (7.69%, 95% CI: 6.88–8.58%) and lowest in the ≥60 years group (3.02%), with statistically significant differences among groups (*p* < 0.001). The age distribution of influenza virus followed a different pattern: the highest rate was in the 6–14 years group (15.17%, 95% CI: 14.18–16.21%) and the lowest in the 0–5 years group (6.15%), also with statistically significant differences (*p* < 0.001). Pairwise comparisons (Bonferroni correction, α = 0.005) showed that the SARS-CoV-2 positive rate in the 15–34 years group was significantly higher than those in the 0–5, 6–14, 35–59 and ≥60 years groups (all *p* < 0.005), and the 35–59 years group was also higher than the ≥60 years group (*p* = 0.0006); the influenza positive rate in the 6–14 years group was significantly higher than those in the 0–5, 35–59 and ≥60 years groups (all *p* < 0.005), with no significant difference from the 15–34 years group (*p* = 0.008).

**Table 1:** Detection of SARS-CoV-2 and influenza virus among ARI cases by demographic characteristics.

Characteristic	Stratum	No. tested	SARS-CoV-2 positive	SARS-CoV-2 rate (%)	95% CI	Influenza positive	Influenza rate (%)	95% CI	*χ^2^*	*p*
Sex	Male	11,788	459	3.89	3.56–4.26	1,140	9.67	9.15–10.22		
	Female	10,648	527	4.95	4.55–5.38	1,065	10.00	9.45–10.59	14.83	<0.001
Age group	0–5 y	6,878	222	3.23	2.84–3.67	423	6.15	5.61–6.74		
	6–14 y	4,807	214	4.45	3.90–5.07	729	15.17	14.18–16.21		
	15–34 y	3,786	291	7.69	6.88–8.58	506	13.37	12.32–14.49		
	35–59 y	2,665	129	4.84	4.09–5.72	260	9.76	8.69–10.94		
	≥60 y	4,300	130	3.02	2.55–3.58	287	6.67	5.97–7.46	140.47	<0.001

**Figure 1:**
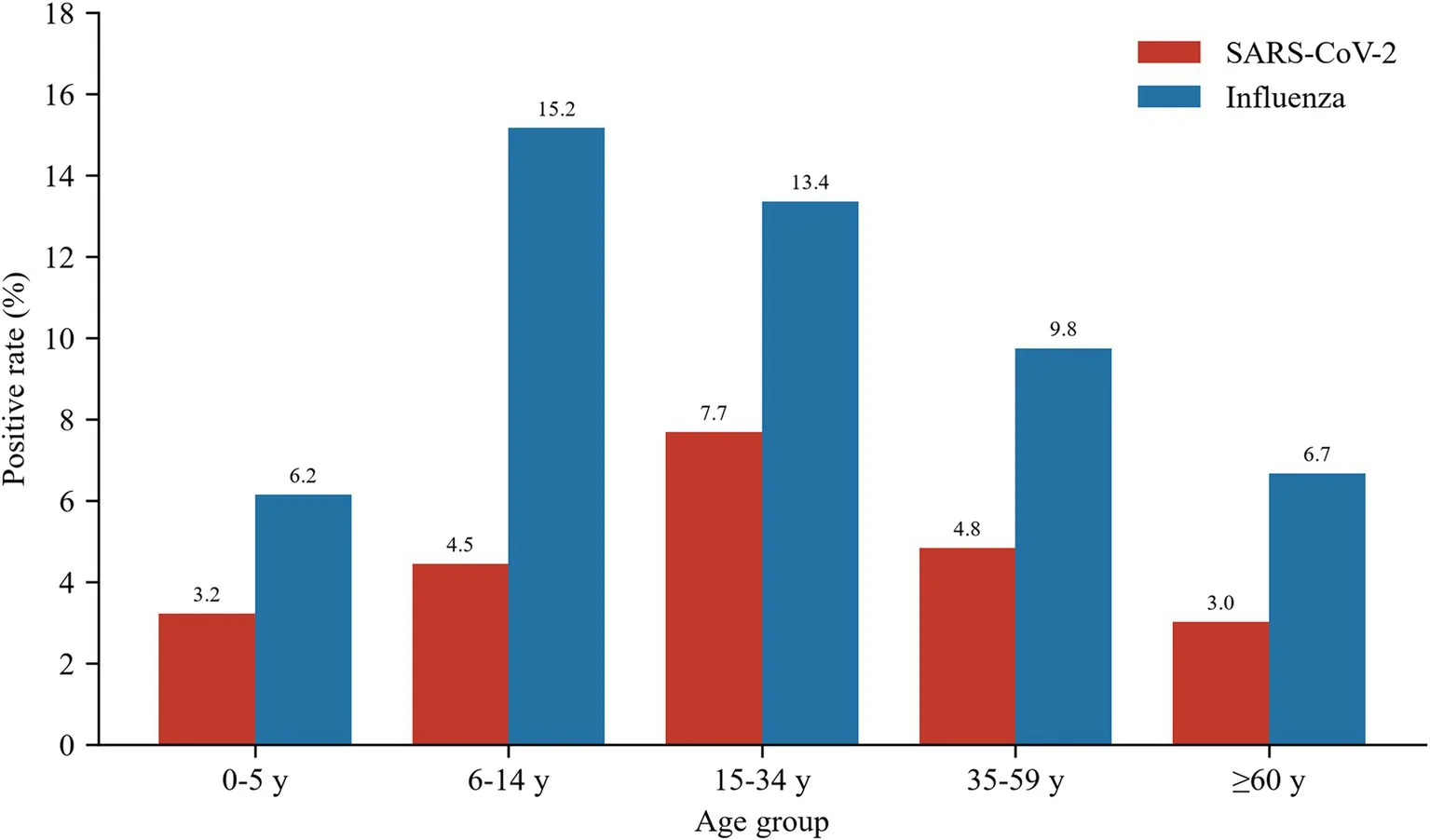
Age-stratified detection rates of SARS-CoV-2 and influenza virus.

### 3.3 Temporal distribution

Monthly observation showed a summer epidemic pattern for SARS-CoV-2 (Figure 2), with monthly positive rates ranging from 0 to 17.46%; two summer peaks occurred in July–August 2024 and May–July 2025, with the highest rate of 17.46% (95% CI: 15.20–19.99%) in June 2025.

**Figure 2:**
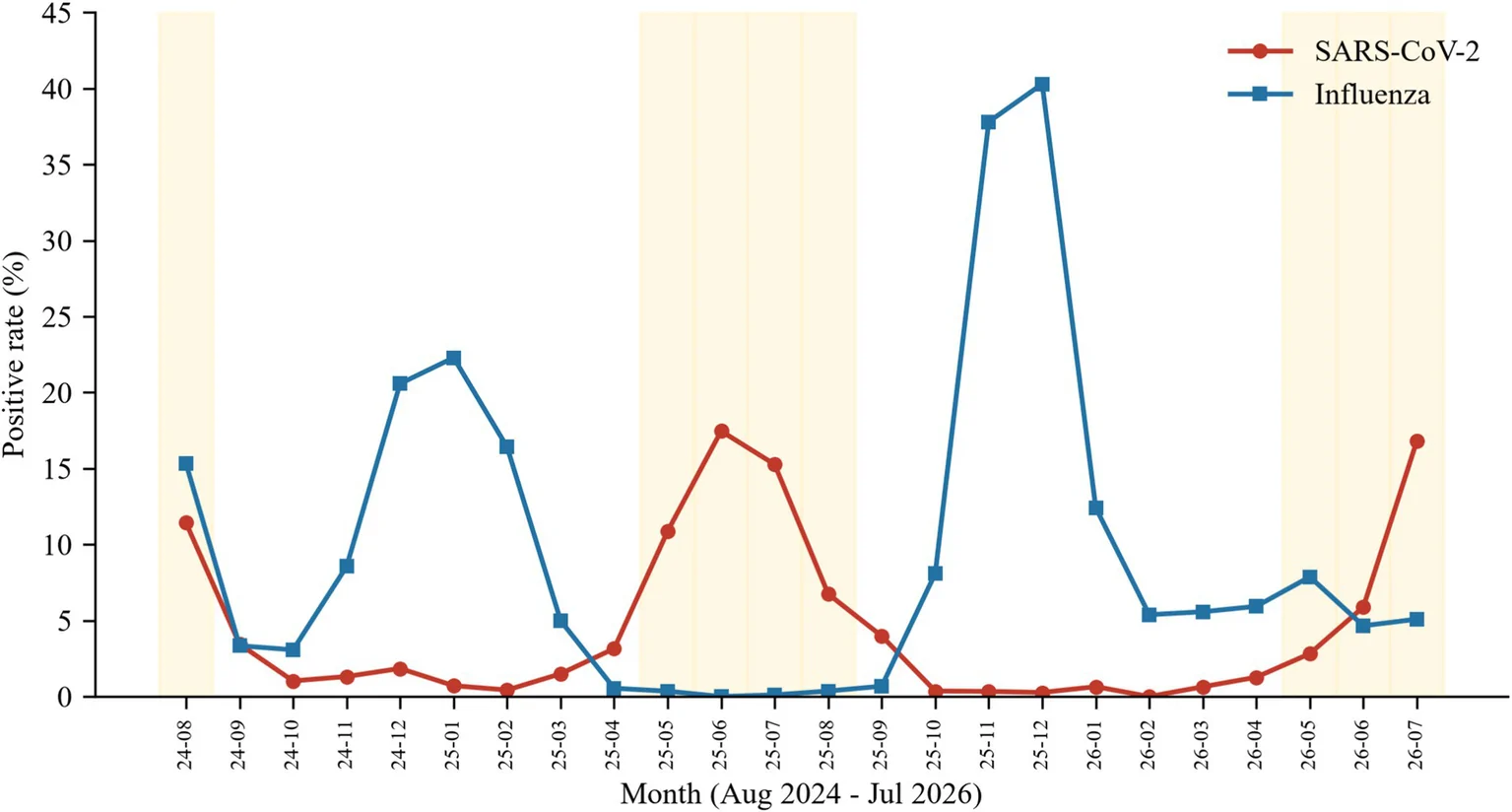
Monthly distribution of SARS-CoV-2 and influenza virus positive rates, August 2024-July 2026.

In contrast, influenza virus showed a typical winter–spring seasonal pattern (Figure 2), with two winter–spring peaks in December 2024 to January 2025 and November to December 2025; the highest rate during the study period was reached in December 2025 (40.30%, 95% CI: 37.47–43.20%).

Coinfections were temporally concentrated in the window when the two viruses circulated simultaneously: 6 cases in August 2024; 2 in December 2025; 1 in May 2026; and 2 in July 2026.

### 3.4 Regional distribution

Positive rates differed markedly across the seven sentinel cities (Table 2; Figure 3). The SARS-CoV-2 positive rate was highest in Zigong (6.03%) and lowest in Chengdu (3.23%), with statistically significant differences among regions (*p* < 0.001); pairwise comparisons (Bonferroni correction, *α* = 0.0024) showed that the rates in Panzhihua and Zigong were significantly higher than those in Chengdu and Mianyang (*p* < 0.0024), with no significant differences among the remaining cities.

**Table 2:** Detection of SARS-CoV-2 and Influenza Virus Across Different Regions.

Region	No. tested	SARS-CoV-2 rate (%)	95% CI	Influenza rate (%)	95% CI	Coinfections	Coinfection rate (%)	95% CI	Fisher *p*
Chengdu	3,124	3.23	2.67–3.91	10.88	9.84–12.02	0	0.00	0.00–0.12	–
Dazhou	3,413	3.87	3.27–4.57	13.07	11.98–14.24	7	0.21	0.10–0.42	<0.001
Nanchong	3,173	4.29	3.63–5.05	6.33	5.54–7.24	0	0.00	0.00–0.12	–
Mianyang	3,116	3.92	3.29–4.65	9.31	8.34–10.38	2	0.06	0.02–0.23	–
Panzhihua	3,255	5.38	4.65–6.21	9.74	8.77–10.81	0	0.00	0.00–0.12	–
Zigong	3,236	6.03	5.26–6.90	12.98	11.86–14.18	2	0.06	0.02–0.23	–
Ya’an	3,119	4.01	3.37–4.75	6.12	5.34–7.02	0	0.00	0.00–0.12	–
Total	22,436	4.39	4.13–4.67	9.83	9.45–10.22	11	0.05	0.03–0.09	–

Fisher’s exact test indicated that only Dazhou had a statistically significantly higher coinfection rate compared with other sentinel cities (*p* < 0.001); pairwise comparisons for other cities yielded no statistically significant differences.

**Figure 3:**
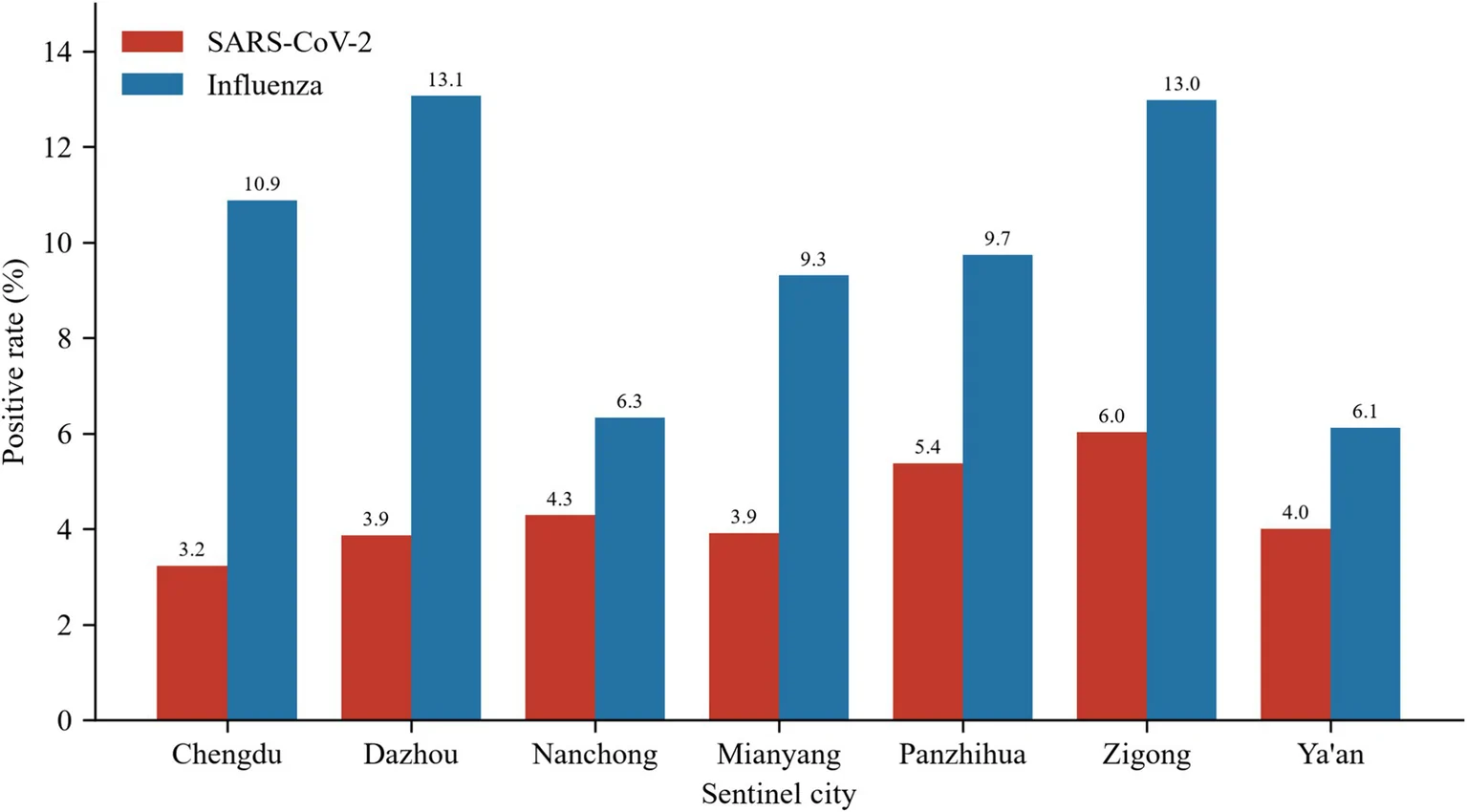
City-specific detection rates of SARS-CoV-2 and influenza virus.

The regional pattern of influenza virus differed: the positive rate was highest in Dazhou (13.07%) and lowest in Nanchong (6.33%), with statistically significant differences among regions (*p* < 0.001); pairwise comparisons showed that the rates in Dazhou and Zigong were significantly higher than those in Nanchong, Ya’an, Mianyang and Panzhihua (*p* < 0.0024), with no significant difference between the two cities.

### 3.5 Multivariable logistic regression analysis

Multivariable logistic regression with SARS-CoV-2 positivity as the outcome showed that summer was an independent risk factor for SARS-CoV-2 infection (OR = 23.88, 95% CI: 17.04–33.46, *p* < 0.001); the 15–34 years group had a higher infection risk than the 0–5 years group (OR = 2.32, 95% CI: 1.87–2.88); male sex was a statistically significant independent protective factor against SARS-CoV-2 infection (male vs. female: OR = 0.86, 95% CI: 0.75–0.98, *p* = 0.027); the ≈14% lower odds in males was modest and of limited clinical significance, while sex showed no association with influenza positivity (*p* = 0.22).

Multivariable logistic regression with influenza positivity as the outcome showed that winter was the reference high-risk season, with significantly lower risks in spring, summer and autumn (summer OR = 0.16, 95% CI: 0.14–0.19); the 6–14 years group had the highest infection risk (OR = 2.52, 95% CI: 2.21–2.88; Table 3).

**Table 3:** Multivariable logistic regression analysis.

Variable	Reference	SARS-CoV-2 OR (95% CI)	*p*	Influenza OR (95% CI)	*p*
Age group	0–5 y (reference)				
6–14 y		1.43 (1.17–1.74)	<0.001	2.52 (2.21–2.88)	<0.001
15–34 y		2.32 (1.87–2.88)	<0.001	2.14 (1.83–2.51)	<0.001
35–59 y		1.32 (1.04–1.69)	0.024	1.70 (1.42–2.02)	<0.001
≥60 y		1.17 (0.92–1.49)	0.20	1.33 (1.13–1.57)	<0.001
Sex	Female (reference)				
Male		0.86 (0.75–0.98)	0.027	1.06 (0.97–1.16)	0.22
Season	Winter (reference)				
Spring		5.56 (3.88–7.96)	<0.001	0.17 (0.15–0.20)	<0.001
Summer		23.88 (17.04–33.46)	<0.001	0.16 (0.14–0.19)	<0.001
Autumn		3.10 (2.11–4.53)	<0.001	0.44 (0.40–0.50)	<0.001
Case type	ILI (reference)				
SARI		0.41 (0.34–0.50)	<0.001	0.52 (0.46–0.58)	<0.001
Region	Nanchong (reference)				
Chengdu		1.14 (0.84–1.56)	0.40	1.86 (1.50–2.30)	<0.001
Dazhou		1.27 (0.96–1.66)	0.09	2.53 (2.09–3.06)	<0.001
Mianyang		1.01 (0.78–1.31)	0.94	1.63 (1.34–1.99)	<0.001
Panzhihua		1.72 (1.34–2.21)	<0.001	1.95 (1.60–2.37)	<0.001
Zigong		1.65 (1.30–2.08)	<0.001	2.37 (1.97–2.85)	<0.001
Ya’an		1.33 (1.00–1.76)	0.050	1.12 (0.89–1.40)	0.33

### 3.6 Influenza virus subtype distribution

Influenza virus-positive cases were predominantly influenza A (1 929 cases, 87.5%, including 1,045 A(H3) and 875 A(H1N1) cases), with 276 influenza B cases (12.5%, predominantly the B/Victoria lineage) (Table 4; Figure 4).

**Table 4:** Subtype distribution of influenza virus among positive cases.

Influenza subtype	No. positive	Proportion (%)
Influenza A	1929	87.5
A(H3)	1,045	47.4
A(H1N1)	875	39.7
Influenza B	276	12.5
B/Victoria	275	12.5
B/Yamagata	0	0.0
Total	2,205	100.0

Proportions were calculated with influenza-positive cases as the denominator.

**Figure 4:**
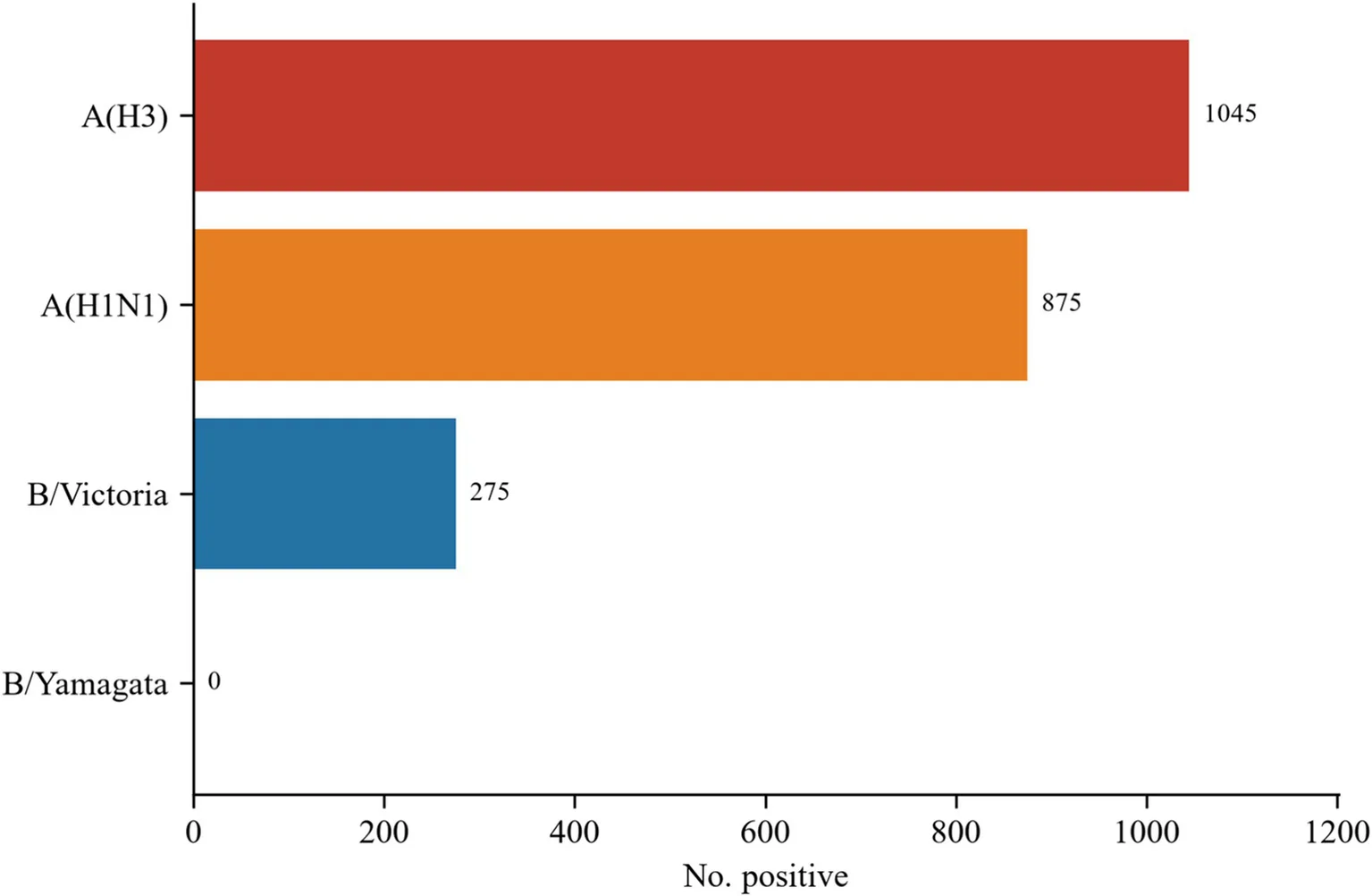
Influenza virus subtype composition.

### 3.7 Coinfection analysis

A total of 11 coinfections of SARS-CoV-2 and influenza virus were detected, with a coinfection rate of 0.05% (95% CI: 0.03–0.09%), accounting for 1.12% of SARS-CoV-2-positive cases and 0.50% of influenza-positive cases. If the two viruses circulated independently, the expected coinfection rate would be the product of the two overall positive rates (4.39% × 9.83% = 0.43%, approximately 97 cases); the observed 11 cases were far below this expectation, with O/E = 0.11 and *p* < 0.001 by the exact binomial test, suggesting a negative (asynchronous) association at the population level rather than synergy.

By epidemic period, only August 2024 (770 cases) met the threshold of both virus positive rates >5% and >10%; 6 coinfections (0.78%) were detected in that month, whereas 5 (0.02%) were detected among 21,666 cases in the asynchronous period, with OR = 34.02 (95% CI: 8.62–141.16; Fisher’s exact test, *p* < 0.001). This difference mainly reflects the time accessibility that coinfection can only occur when the two viruses circulate simultaneously; even in the concurrent month, the observed coinfections remained below the independent expectation (approximately 13 cases). Regionally, 7 of the 11 coinfections (63.6%) were detected in Dazhou, with a coinfection rate of 0.21% significantly higher than other regions (Fisher’s exact test, *p* < 0.001); in August 2024, the SARS-CoV-2 and influenza positive rates in Dazhou were 14.69 and 39.86%, respectively, both at high levels, suggesting local concurrent circulation. Given the small number of coinfection events and their strong spatiotemporal clustering, specimen-submission-driven or testing-batch-related clustering cannot be fully excluded, and verification with larger samples is warranted.

Among the 11 coinfection cases, 9 (81.8%) were influenza-like illness (ILI) cases and 2 (18.2%) were hospitalized SARI cases; by month, there were 6 cases in August 2024 (4 in Dazhou and 2 in Zigong), 2 in December 2025 (both in Dazhou), 1 in May 2026 (Mianyang) and 2 in July 2026 (1 each in Dazhou and Mianyang). The case-by-case distribution by case type, region and month is shown in Table 5.

**Table 5:** Case type, region and time distribution of the 11 SARS-CoV-2 and influenza virus coinfection cases.

No.	Case type	Region	Month of collection	Sex	Age (years)	Influenza subtype
1	ILI	Dazhou	2024-08	Female	55	A(H1N1)pdm09
2	ILI	Dazhou	2024-08	Female	6	A(H1N1)pdm09
3	ILI	Zigong	2024-08	Male	54	A(H1N1)pdm09
4	ILI	Dazhou	2024-08	Male	84	A(H1N1)pdm09
5	ILI	Dazhou	2024-08	Female	37	A(H1N1)pdm09
6	ILI	Zigong	2024-08	Male	71	A(H1N1)pdm09
7	ILI	Dazhou	2025-12	Female	26	A(H3)
8	ILI	Dazhou	2025-12	Female	15	A(H3)
9	SARI	Mianyang	2026-05	Male	1	B/Victoria
10	SARI	Dazhou	2026-07	Male	1	A(H1N1)pdm09
11	ILI	Mianyang	2026-07	Female	24	A(H3)

Month refers to the specimen collection time; coinfection indicates simultaneous detection of SARS-CoV-2 and influenza virus nucleic acid in the same specimen.

## 4 Discussion

Based on data from 22,436 ARI cases monitored over 24 consecutive months (August 2024–July 2026) at seven national respiratory pathogen sentinel hospitals in Sichuan Province, the main findings were: overall positive rates of 4.39% for SARS-CoV-2 and 9.83% for influenza virus, a coinfection rate of only 0.05%, and a significant negative (asynchronous) association (O/E = 0.11).

SARS-CoV-2 showed a summer epidemic pattern, peaking in June 2025 (17.46%), and summer was an independent risk factor (OR = 23.88, 95% CI: 17.04–33.46, *p* < 0.001), consistent with the national pattern of off-season circulation (13); several regions of China also reported changes in the circulation rhythm of the two viruses over the same period: influenza-like illness surveillance in Inner Mongolia in 2023 showed that the epidemic peaks of the two viruses were staggered and alternated (14), surveillance in Jilin Province in 2023–2024 reported that influenza virus still peaked in winter–spring (15), surveillance in Guilin in 2023–2024 showed co-circulation of influenza with respiratory syncytial virus and other pathogens, with detections also in summer (16), and dynamic surveillance in Tongzhou District, Beijing, 2021–2024, observed marked changes in influenza activity intensity and timing compared with the pre-pandemic period (17). This suggests that the summer resurgence of COVID-19 is not unique to Sichuan. Unlike northern provinces (Inner Mongolia, Jilin) where influenza still mainly peaked in winter–spring (14, 15), SARS-CoV-2 in Sichuan showed a summer-dominant pattern; the difference in rhythm may relate to latitude and climate (cold, dry winters in the north favour influenza transmission) and to population immunity background (repeated summer SARS-CoV-2 circulation in southwest China). The summer peak can be explained by the humid subtropical climate of the Sichuan Basin: high temperature and humidity suppress transmission of enveloped viruses such as influenza but do not markedly weaken SARS-CoV-2, while waning population immunity over time, combined with summer holiday mobility and gathering, jointly contributed to the summer resurgence of COVID-19 (10). The COVID-19 pandemic and its non-pharmaceutical interventions have profoundly changed the seasonality and co-circulation patterns of respiratory viruses globally, with many viruses showing off-season circulation and peak reshaping after interventions were lifted (18); the single summer peak observed in Sichuan should be understood against this background of global rhythm reconstruction.

Influenza virus showed a typical winter–spring epidemic pattern, peaking in December 2025 (40.30%), with significantly lower risk in spring, summer and autumn (summer OR = 0.16) (13, 15, 19). In terms of population, SARS-CoV-2 was highest in the 15–34 years group (7.69%), a group with frequent social contact and relatively low booster vaccination coverage; influenza risk was highest among school-aged children aged 6–14 years (OR = 2.52), related to closed school settings (9). The 0–5 years group had the lowest rate; apart from milder illness in young children, this more likely reflects underestimation due to low health-care-seeking rates and limited sampling compliance, indicating a need to strengthen active surveillance in childcare and community settings. In terms of subtype composition, influenza-positive cases in this study were predominantly influenza A (87.5%), with similar detection numbers for A(H3) and A(H1N1)pdm09 (1,045 vs. 875), and influenza B accounting for only 12.5%, predominantly the B/Victoria lineage, consistent with the national pattern in which influenza A predominated and B/Victoria was dominant (15). The alternating predominance of H3 and H1N1 within influenza A suggests subtype turnover of circulating strains; selection of seasonal influenza vaccine components should continue to be based on subtype surveillance results, and dynamic subtype-level surveillance and vaccine matching assessment are recommended. Notably, the B/Yamagata lineage has been rarely or not detected globally in recent years (20), consistent with its absence over 24 consecutive months in this study; however, as an observation from a single province over 24 months, this is insufficient to infer local extinction of the lineage, and continued surveillance remains necessary to inform vaccine composition decisions.

The coinfection rate (0.05%) was significantly lower than the independent expectation (0.43%, O/E = 0.11, *p* < 0.001), indicating an off-peak negative association at the population level (21). Multiple systematic reviews have shown that the pooled prevalence of influenza coinfection among confirmed COVID-19 cases is approximately 2.45%; although overall infrequent, coinfected patients have an increased risk of severe disease and death (22, 23, 36). The observation units in this study were all ARI patients attending outpatient and emergency ILI services and inpatient SARI services, so the coinfection rate was lower than in studies based mainly on hospitalized confirmed COVID-19 patients and better reflects the true exposure pattern at the population level. This negative association may arise at three levels: at the cellular level, cross-inhibition of innate immunity and interferon pathways between viruses can reduce the probability of dual infection (24–26); at the population level, staggered epidemic peaks reduce opportunities for co-exposure and are the main source of the negative association; at the behavioural level, home isolation and reduced health-care seeking after infection further reduce dual exposure (27). Notably, *in vitro* experiments have shown that coinfection with influenza A virus can enhance SARS-CoV-2 infectivity (28), which does not contradict the population surveillance results: the former reflects enhanced infectivity at the single-cell level, whereas the latter is constrained by co-exposure opportunities; the two operate at different scales, and *in vitro* evidence should not be used to directly infer elevated population-level risk. Because this is an ecological, population-level surveillance analysis, the observed O/E ratio cannot by itself distinguish a population-level temporal mismatch in co-exposure opportunity from genuine individual-level viral interference; separating these two mechanisms requires virological or prospective cohort studies.

Viral interference provides biological plausibility for the above negative association: infection with one respiratory virus can induce innate immune responses such as interferons, creating a transient antiviral state in target tissues and thereby reducing the probability of subsequent infection by the same or a heterologous virus (24, 25); mutual inhibition between rhinovirus and influenza virus has been repeatedly demonstrated in experimental and population studies. However, inferring pathogen-pathogen interactions from coinfection prevalence data has methodological limitations: when multiple pathogens co-circulate and testing resources are limited, coinfection proportions can be systematically underestimated, and test-negative designs may also misjudge the direction of interaction (26). This study used a unified multiplex nucleic acid testing platform to detect both viral targets simultaneously in all included cases, avoiding the detection bias caused by prioritising a single pathogen; therefore, the negative association of O/E = 0.11 mainly reflects genuine temporal separation of circulation rather than differences in testing strategy. The multiplex platform detects all targets simultaneously in discrete amplification channels, so the high carriage of bacteria such as *Haemophilus influenzae* (15.59%) and *Streptococcus pneumoniae* (10.98%)—which largely reflects colonisation rather than acute infection—does not competitively suppress SARS-CoV-2 or influenza detection; nevertheless, because the analysis focused on the two viruses, potential interactions with these co-detected pathogens were not characterised.

Regional distribution showed geographical heterogeneity consistent with national multi-pathogen surveillance (9, 29): Panzhihua, located in a dry-hot valley with a warm, dry climate, had relatively weak winter influenza activity, similar to the pathogen spectrum of subtropical cities such as Guilin (16); Dazhou in northeastern Sichuan is a major labour-exporting city with frequent population movement, and its local SARS-CoV-2 and influenza co-circulation is consistent with reports from Inner Mongolia (14) and Tongzhou, Beijing (17) and other areas with active population movement (30); Zigong in southern Sichuan has dense primary and secondary schools with high child aggregation risk, and the high influenza burden among those aged 6–14 years is consistent with Jilin (15) and the national age distribution of acute respiratory infections (9). After non-pharmaceutical interventions were lifted, detection rates of respiratory pathogens among children rose markedly in many regions of China, with changes in epidemic season and susceptible age, reflecting an immunity gap effect (31–33), which fits with the high influenza burden among school-aged children observed in this study. The overall positive rates of both viruses were higher in outpatient and emergency ILI than in inpatient SARI (SARI OR = 0.41/0.52), partly reflecting inpatient sampling bias, and should be interpreted in combination with SARI surveillance.

Layered prevention and control according to population and regional characteristics is recommended: in summer (May to August), focus health education and venue ventilation on the 15–34 years population, and prioritise SARS-CoV-2 testing for influenza-like illness in fever clinics; in winter–spring, incorporate influenza vaccination for school-aged children aged 6–14 years into routine school arrangements and progressively increase coverage, with implementation of morning and afternoon health checks and absence-for-illness registration; in areas with high coinfection incidence such as Dazhou and Zigong, incorporate combined SARS-CoV-2 and influenza dual-pathogen testing into routine sentinel screening, and test both viruses simultaneously in hospitalized severe cases. Combining serological immunity data with pathogen surveillance can quantify population susceptibility and improve prediction of epidemic timing and intensity, helping to enhance the forward-looking early-warning capacity of sentinel surveillance (34).

## 5 Limitations

First, the study relied on only seven urban tertiary (grade A) sentinel hospitals, without coverage of community and asymptomatic populations, resulting in health-care-seeking selection bias; if community-level staggering of the two viruses is stronger, the true coinfection rate may be lower than observed, but if the care-seeking population is more concentrated among high-risk individuals, the observed value may also be overestimated, so extrapolation to the whole province requires caution (18, 34). In addition, because no province-wide pre-pandemic (2015–2019) dual-pathogen sentinel surveillance exists for Sichuan, the present study could not quantitatively compare the observed rhythm with a historical baseline; such a comparison is reserved for future analyses with access to pre-pandemic sentinel data. Second, the analysis focused on SARS-CoV-2 and influenza and did not include coinfection combinations with respiratory syncytial virus, adenovirus, *Mycoplasma pneumoniae* and other pathogens, potentially underestimating the multi-pathogen infection burden; existing studies have shown a significant increase in multi-pathogen coinfection rates among children after non-pharmaceutical interventions were lifted (31, 32), indicating that the dual-pathogen design of this study remains insufficient to characterise multi-pathogen infection. Third, data on vaccination history, underlying diseases and clinical outcomes were lacking, precluding quantification of coinfection severity and vaccine protection. Future research should expand coverage of community and primary-care sentinel sites, conduct full-spectrum respiratory multi-pathogen coinfection analysis, add clinical outcome follow-up, and incorporate meteorological, population mobility and population immunity factors (33–35) into the analysis.

## 6 Conclusion

From August 2024 to July 2026, the two viruses in Sichuan Province showed marked seasonal stagger: SARS-CoV-2 mainly circulated in summer (June 2025) and influenza virus in winter–spring (December 2025); high-risk populations were those aged 15–34 years for SARS-CoV-2 and 6–14 years for influenza. Coinfection was uncommon and significantly lower than the independent expectation, with no synergistic enhancement at the population level, but cases were highly concentrated in Dazhou and in a few months. Layered prevention and control according to pathogen and regional characteristics is recommended, with emphasis on dual-pathogen screening in key populations and early diagnosis of severe cases.

## Data Availability

The raw data supporting the conclusions of this article will be made available by the authors, without undue reservation.
